# An In Silico Investigation of Brazilian Green Propolis Extracts as Potential Treatment for COVID-19

**DOI:** 10.1021/acsomega.5c02121

**Published:** 2025-08-05

**Authors:** Jeronimo Geraldo Ferreira Júnior, Janaína Brandão Seibert, Viviane Martins Rebello dos Santos, Lucas Resende Dutra Sousa, Tatiane Roquete Amparo, Thalita Marcolan Valverde, Vagner Rodrigues Santos, Junnia Alvarenga de Carvalho Oliveira, Paula Melo de Abreu Vieira, Vagner de Oliveira Machado, Ângela Leão Andrade

**Affiliations:** † Departamento de Química, 28115 Universidade Federal de Ouro Preto, Ouro Preto 35400-000, Brasil; ‡ Departamento de Química, Universidade Federal de São Carlos, São Carlos 13565-905, Brasil; § Departamento de Farmácia, Universidade Federal de Ouro Preto, Ouro Preto 35400-000, Brasil; ∥ Departamento de Morfologia, 28114 Universidade Federal de Minas Gerais, Belo Horizonte 31270-901, Brazil; ⊥ Departamento de Clínica, Patologia e Cirurgias Odontológicas, Universidade Federal de Minas Gerais, Belo Horizonte 31270-901, Brazil; # Departamento de Microbiologia, Universidade Federal de Minas Gerais, Belo Horizonte 31270-901, Brazil; ¶ Departamento de Ciências Biológicas, Universidade Federal de Ouro Preto, Ouro Preto 35400-000, Brasil; ∇ Núcleo Tecnológico EPAMIG Uva e Vinho, Fazenda Experimental de Caldas, Empresa de Pesquisa Agropecuária de Minas Gerais, Caldas 37780-000, Brasil

## Abstract

The absence of a specific antiviral treatment for the highly virulent and lethal coronavirus disease 2019 (Covid-19) remains a significant challenge. The potential of natural agents with immune-promotion capabilities, including apitherapy products, is being investigated as a potential therapeutic modality. Propolis is characterized by a high concentration of bioactive compounds, which exhibit significant antimicrobial, bactericidal, antiviral, anti-inflammatory, immunomodulatory, and antioxidant properties. This study investigated the potential of ethanolic extracts of Brazilian green propolis for COVID-19 treatment. Propolis extracts were obtained at room (EEPV-F) and under 70 °C-heat (EEPV-Q) temperatures, and the extraction yields were 36.74% and 53.54%, respectively. Total flavonoid contents (10.6 ± 0.9 and 6.6 ± 0.2 mg QE g^–1^), total phenolic compounds (44 ± 2 and 66 ± 3 mg GAE g^–1^), and antioxidant capacity by DPPH (IC_50_ values of 18.0 ± 0.3 and 16.6 ± 0.5 μg mL^–1^) and ABTS (IC_50_ values of 16.6 ± 0.02 and 15.3 ± 0.2 μg mL^–1^) assays were determined. The extracts were not cytotoxic to RAW 264.7 macrophages and exhibited dose-dependent anti-inflammatory activity, particularly EEPV-Q. All EEPV-Q concentrations significantly reduced nitric oxide production and demonstrated suitability for further investigation. Molecular docking analysis revealed that the compound guibourtinidol-(4alpha→8)-epiafzelechin exhibited stronger binding affinity for PLpro, RdRp, and Spike-analyzed enzymes. Both EEPV-F and EEPV-Q presented hemolytic activity below 5% across all tested concentrations. Given the limited therapeutic options available for COVID-19, the utilization of green propolis as a therapeutic agent has emerged as a promising and relevant approach. This natural product possesses several advantages, including its safety, ease of oral administration, and its availability as both a natural supplement and a functional food.

## Graphical abstract

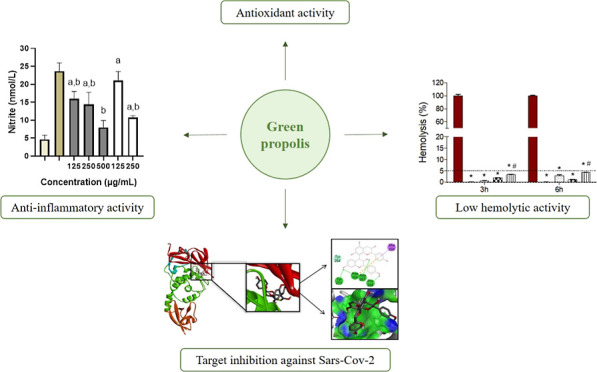

## 1 Introduction

Viral outbreaks are a frequent concern for populations.^1–4^ Among the viral diseases, COVID-19 stands out as much more deadly than influenza and other diseases that have recently had a global impact.^5^ The infection process of SARS-CoV-2, the virus that causes the disease known as “Coronavirus Disease 2019” (henceforth termed ″Covid-19″), is characterized by the binding of the viral proteins spikes to the angiotensin-converting enzyme 2 receptor.^6^ The activation of the protein spike is mediated by proteases, such as the transmembrane protease serine type 2 (TMPRSS2), which plays an important role in viral infection.^7^ Following entry and endocytosis, the infection of the coronavirus has been shown to induce positive regulation of PAK1, a serine/threonine kinase that has been demonstrated to mediate lung inflammation, pulmonary fibrosis, and other critical mortality factors. It has been demonstrated that an augmentation in PAK1 levels has the capacity to impede the adaptive immune response, thus engendering conditions conducive to viral replication.^8,9^

The presence of SARS-CoV-2 infection has been demonstrated to be associated with elevated levels of chemokines and activated pro-inflammatory cytokines. This has been shown to result in the development of atypical pneumonia, which is characterized by rapid respiratory compromise and pulmonary failure.^10^ Immunological and inflammatory phenomena (e.g., cytokine release syndrome) have been demonstrated to be significant components of the SARS-CoV-2 infection spectrum. It has been demonstrated that these mechanisms are more closely associated with organ dysfunction than that with the viral load itself.^11^ In this setting, a retrospective observational study identified elevated serum levels of pro-inflammatory cytokines, including IL-6, IL-1, and TNF-α, in patients with severe cases of COVD-19, in comparison with individuals manifesting mild symptoms.^12^

A diverse array of molecules and pharmaceutical agents have been employed in the treatment of viral diseases, including interferon-α, ribavirin, cidofovir, acyclovir, and ganciclovir, among others, which have demonstrated efficacy in the management of such manifestations.^13,14^ Nevertheless, it should be noted that the safety and efficacy of these medications are not guaranteed, and there is a possibility of adverse effects arising, including, but not limited to, kidney injury,^15^ neurological damage,^16^ and others. The mixture of antibiotics to treat viral respiratory diseases has been reported to cause further issues, primarily bacterial resistance to these medications.^17^

It is evident that natural products have a long history as preventative measures and remedies for a wide range of ailments^18,19^ are considered among the options for adjuvant treatment of SARS-CoV-2 infection^8^ due to their general affordability and low risk of undesirable side effects. Furthermore, some natural products have demonstrated antiviral activity^20,21^ and have also been investigated as potential therapeutic candidates against COVID-19, as discussed in recent reviews on small-molecule drug development and repositioning strategies for SARS-CoV-2 infection.^22^ In parallel, increasing attention has been given to the broader biological activities of natural products, such as propolis, which have demonstrated immunomodulatory properties in various contexts. Notably, Zhou et al.^23^ discussed the potential of propolis in conditions associated with allergic inflammation, further highlighting its versatility as a therapeutic agent. Given the central role of immune dysregulation in the pathophysiology of COVID-19, particularly in individuals with pre-existing inflammatory or allergic conditions, these findings reinforce the rationale for investigating propolis and other natural products as potential adjuvant therapies in viral infections.

Propolis, a resinous complex mixture produced by bees of the species *Apis mellifera*, has been used by humans since ancient times for its medicinal attributes. Propolis is widely used by bees for hive protection, preventing infiltration of water, putrefaction of dead intruders, and maintaining local asepsis.^24,25^ The substance under scrutiny is a highly complex mixture containing over 300 chemical compounds, some of which have been documented to have beneficial health outcomes. The chemical composition of propolis is diverse, with resins, waxes, essential oils, pollen, and a variety of organic compounds being the primary components. These include phenolic compounds, flavonoids, terpenes, esters, aldehydes, alcohols, aromatic compounds, and specific antioxidant compounds, such as beta-carotene, acid caffeic, and kaempferol.^26^ The diversity of the biochemical composition of propolis is a consequence of a number of factors, including geographical origin, climate, and water availability.^27,28^ It has been established that all types of propolis possess a range of biological activities, including antimicrobial, antineoplastic, anti-inflammatory, antioxidant, hepatoprotective, cariostatic, and immunostimulatory properties.^28^

The use of propolis as a raw material is not a viable option and must be purified by extraction with solvents.^25^ The antiviral properties of extracts of temperate-climate propolis have been demonstrated in a range of studies. It has been demonstrated that the extracts exhibit a high level of antiviral activity against a range of viruses, covering a broad spectrum, whether it is alcoholic or aqueous.^27–30^

Although the antibacterial properties of propolis extracts have been widely studied, their antiviral potential remains relatively underexplored.

The objective of the present study was to undertake a chemical characterization of green propolis extracts obtained at two different temperatures, with a view to identifying compounds with therapeutic potential for the treatment of patients infected with the SARS-CoV-2 virus. The specific objectives included: (i) obtaining ethanolic extracts of green propolis; (ii) assessing their *in vitro* antioxidant capacity, total phenols and flavonoids content, cytotoxicity, hemolytic activity, and anti-inflammatory potential; (iii) chemically characterize the obtained ethanolic extracts; (iv) indicate the anti-inflammatory action mechanism in silico of some of the identified compounds; and (v) evaluate the anti-SARS-CoV-2 in silico potential of the compounds that exhibit anti-inflammatory action.

## 2 Experimental Section

### 2.1 Natural Product and Chemicals Reagents

The raw samples of green propolis were obtained in Caeté, in the state of Minas Gerais, located in the Southeast Region of Brazil (SL 195248 and WL 434011), kindly provided by Pharma Néctar. 2,2-diphenyl-1-picrylhydrazyl (DPPH); Folin-phenol reagent Ciocalteu; sodium bicarbonate; aluminum chloride; potassium persulfate; 2, 2′-azino- bis (3-ethylbenzothiazoline-6-sulfonic acid) (ABTS); quercetin; gallic acid; interferon γ (IFN-γ); lipopolysaccharide (LPS); sodium nitrite; 3–4,5-dimethylthiazol-2-bromide il-2,5-diphenyltetrazolium (MTT); and RPMI 1640 medium were purchased from Sigma-Aldrich. The 70% ethanol was purchased from Araucária. Dimethyl sulfoxide was bought from Synth. All chemicals were utilized in an unpurified state.

### 2.2 Preparation of 70% Ethanolic Extracts of Green Propolis at Room Temperature or under Heat (EEPV-F or EEPV-Q, Respectively)

A total of 0.5000 g of raw green propolis powder was mixed with 150 mL of 70% ethanol, and a magnetic stirrer was used for 48 h at room temperature or in a water bath at 70 °C. Subsequently, the separation of the insoluble fraction was conducted through the process of filtration. The resultant filtrate was then collected in a flask. The solvent was evaporated using a rotary evaporator to obtain the dry ethanolic extract of the green propolis. This dry extract was heavy, and the yield was calculated, with the result expressed as the percentage of weight of the crude extract in relation to that of the raw material. Samples were stored at room temperature. Extracts obtained at room temperature and at hot temperature were labeled as EEPV-F and EEPV-Q, respectively.

### 2.3 Evaluation of Antioxidant Activity

The antioxidant activity was assessed by *in vitro* photocolorimetric methods, utilizing the free radicals 2,2-diphenyl-1-picrylhydrazyl (DPPH^•^) and 2,2′-azinobis-3-ethylbenzothiazoline-6-sulfonic (ABTS^•+^). The scavenging activity of phenolic compounds against the DPPH^•^ free radical was evaluated following Ferri et al.^31^ with some modifications. The main difference between the method developed by Ferri et al. and the approach adopted in the present study is that in the former, the pH of the reaction medium was carefully controlled between 4 and 8, and the concentration of the DPPH solution was adjusted according to the characteristics of each sample. Conversely, in the present study, the pH level was not controlled, and a fixed DPPH solution at 0.008% (w/v) in ethanol was employed throughout the assays. In summary, a stock solution of DPPH^•^ at 0.008% (w/v) in ethanol was prepared. The extracts and quercetin (standard) were solubilized in ethanol to obtain stock solutions of 200.0 and 400.0 μg mL^–1^, respectively. Aliquots of the substance were then dispensed in order to yield final solutions of 2.3 to 92 μg mL^–1^ of extracts. Then, a volume of 1250 μL of DPPH^•^ solution was added to each of these samples. The final volume was adjusted to 3000 μL with ethanol. The negative control was obtained by mixing 1250 μL of the DPPH^•^ with 1750 μL of ethanol, which was used to calculate the percentage of inhibition of free radicals. The mixture was stirred and left to stand at room temperature (25 ± 2 °C) in the dark for 30 min, and the absorbance (A) was read at a wavelength of 518 nm in a spectrophotometer using ethanol as a blank. This experiment was carried out three times, and the percentage of DPPH^•^ inhibition of the samples was subsequently calculated as

1 $$\%inhibition=\left(1-{A/A}_{0}\right)\times 100$$

where *A*_0_ is the absorbance at 518 nm DPPH spectrum was obtained without the presence of a sample, and *A* denotes the measured absorption at 518 nm of the reaction mixture comprising DPPH^•^ and the sample under investigation. The IC_50_, defined as the concentration that yields 50% inhibition of DPPH radicals, was determined by plotting the I % (percentage of inhibition) against the respective extract concentration.

The antioxidant activity using the ABTS^•+^ radical was performed according to Re et al.^32^ with modifications. In the method described by Re et al., 7 mM ABTS is mixed with 2.45 mM potassium persulfate (K_2_S_2_O_8_) in aqueous solution. However, in the present study, 7.4 mM ABTS was mixed with 2.6 mM potassium persulfate (K_2_S_2_O_8_), also in aqueous solution. These concentrations were selected based on preliminary tests to optimize the stability and reproducibility of the ABTS^•+^ radical generation under our experimental conditions. In both cases, the mixture was kept in the dark for 12–16 h prior to use. The mixture was kept in the dark for this period of time at room temperature to ensure the complete formation of the ABTS^•+^ radical and to prevent photodegradation, which can compromise the stability and reactivity of the radical cation. The dark incubation period allows for consistent radical generation, resulting in a stable absorbance value suitable for antioxidant activity measurements.^33,34^ Upon the day of analysis, the solution was subjected to dilution with ethanol, yielding an optical density of 0.70 ± 0.02 at 650 nm. The extracts and quercetin standard were dissolved in ethanol to yield stock solutions of 200.0 and 400.0 μg/mL, respectively. Different aliquots were pipetted to give final solutions of 1.0 to 20 μg mL^–1^ of extracts. Subsequently, 1600 μL of ABTS^•+^ were incorporated into these samples. The final volume was adjusted to 2000 μL with the addition of ethanol. The negative control was obtained from 1600 μL of ABTS^•+^ and 400 μL of ethanol. Subsequently, all samples were subjected to an incubation period of 6 min at a temperature of 25 ± 2 °C, with the samples being protected from exposure to light during this time. As previously outlined, the readings were taken at 650 nm, and the percentage of sample sequestration and the median effective concentration (EC_50_) were calculated.

### 2.4 Determination of the Total Phenolic and Flavonoid Content

The method employed in the present study for the determination of total phenolic content was adapted from the Folin-Ciocalteu colorimetric assay, with some modifications when compared to the procedure described by Bonoli et al.^35^ In Bonoli’s method, a larger volume of sample solution was used, followed by the addition of water, Folin-Ciocalteu reagent and aqueous sodium carbonate, with the final reaction mixture incubated for 90 min and absorbance measured at 750 nm. In contrast, the present study utilized smaller volumes of reagents and samples, a slightly higher wavelength for absorbance reading (765 nm), and an extended incubation time of 2 h to ensure full color development. These adjustments were made to suit the specific characteristics of the ethanol extracts of green propolis evaluated in this work while maintaining the fundamental principles of the original assay. Briefly, 800 μL of the stock solution of EEPV-F or EEPV-Q (200.0 μg mL^–1^) were mixed with 600 μL of distilled water and 100 μL of Folin-Ciocalteu reagent. Subsequently, the mixture underwent a one min shaking process, after which it was neutralized by the addition of 400 μL of 15% aqueous sodium bicarbonate. The mixture was then subjected to an additional 30 s of agitation, followed by the introduction of 100 μL of water. Following a 2 h incubation period, the absorbances were measured at a wavelength of 765 nm, employing a spectrophotometer. Gallic acid (3–16 mg L^–1^; *r* ^2^ = 0.9964; *y* = 0.0858*x* + 0.0143) was used as a standard reference and the results were expressed in mg of gallic acid equivalents (GAE) per gram of crude extract (mg GAE g^–1^ of crude extract). The ethanol solution was used as a blank. The experiment was performed on thrice.

The total flavonoid content was determined according to Andrade et al.^36^ A quantity of 750 μL of stock solution of EEPV-F or EEPV-Q was placed in a beaker with 1500 μL of 2% AlCl_3_ in ethanol. The absorbance was determined at 420 nm against 750 μL of propolis extract in 1500 μL of ethanol after 10 min. The quantification of total flavonoids was conducted by means of a standard calibration curve of quercetin (0.5–14 mg L^–1^; *r* ^2^ = 0.9992; *y* = 0.2759*x* – 0.0072). The experiment was conducted on three occasions, and the results were expressed in milligrams of quercetin equivalents (QE) per gram of crude extract (mg of QE g^–1^ of crude extract).

### 2.5 Biological Assay

#### 2.5.1 Hemolysis Assay

A hemolysis assay was performed following the protocol described by Martinez-Rodriguez et al.,^37^ with slight modifications in order to evaluate the hemocompatibility of the samples. This assay quantifies the rupture of erythrocyte membranes upon exposure to the test samples, measuring the release of hemoglobin into the supernatant. This assay was performed using defibrinated sheep blood (Dsyslab). The blood was washed by centrifuging at 4500 rpm for 5 min to remove plasma and buffy coat. The erythrocyte pellet was subjected to a washing procedure comprising three cycles of 0.9% NaCl at 4 °C, followed by centrifugation at 4500 rpm for 5 min. Subsequently, the erythrocytes were diluted in 0.9% sodium chloride (3:11).

Then, 100 μL of erythrocytes were incubated with 900 μL of EEPV-F or EEPV-Q samples at different concentrations (100 and 250 μg mL^–1^) for 3 and 6 h at 37 °C. After incubation, samples were centrifuged (4500 rpm, 5 min, 4 °C), and 100 μL of supernatant were transferred to a 96-well plate. Absorbance was measured at 540 nm using an ELISA reader. Erythrocytes incubated with 0.9% NaCl were used as negative controls. In the present study, the positive controls comprised of erythrocytes that were lysed using either sterile deionized water, with the objective of assessing the occurrence of hemolysis. The experimental triplicate readings were then analyzed.

Hemolysis was calculated with the formula^38^

2 $$Hemolysis\left(\%\right)=\frac{sample\;absorbance-negative\;control}{positive\;control-negative\;control}\times 100$$

The initial hemolysis should not exceed 5%.

#### 2.5.2 Cell Culture

The murine macrophage cell line (RAW 264.7) was cultured in a basal medium containing RPMI (Sigma-Aldrich) supplemented with 10% fetal bovine serum (Gibco) and gentamicin at a final concentration of 50 μg mL^–1^ (Thermo Fisher Scientific). The cells were then subjected to an incubation in a humidified atmosphere containing 5% CO_2_ at a temperature of 37 °C. The subcultures were performed at a ratio of 1:3, and the culture medium was renewed every 2 to 3 days.

#### 2.5.3 Cell Viability

The assessment of cell viability was conducted for RAW 264.7 macrophages subsequent to treatment with EEPV-F and EEPV-Q samples through the utilization of the MTT assay.^39^ The MTT assay is a quantitative method that quantifies mitochondrial metabolic activity, thereby serving as an indicator of viable cells. RAW 264.7 macrophages were cultured in a supplemented RPMI 1640 medium. After cell growth and expansion, 100 μL of the stock solution (5 × 10^5^ cells mL^–1^) were distributed per well in a 96-well microtiter plate, which was incubated at 37 °C with 5% CO_2_ for a twenty-4 h period. The cells were subjected to treatment with samples EEPV-F or EEPV-Q, which were dissolved in RPMI and 2% dimethyl sulfoxide (DMSO), at concentrations of 125, 250, and 500 μg/mL, for a period of 24 h at a temperature of 37 °C with 5% CO_2_. For this procedure, the medium was removed, and the wells were thoroughly washed with RPMI. Subsequently, 100 μL of RPMI devoid of phenol red, comprising 10% bovine fetal serum and 50 μL of filtered 2 mg mL^–1^ MTT, was added to the wells containing the samples. The plates were then covered and left to incubate for a period of 4 h. Subsequent to this interval, the reaction was terminated with 100 μL of DMSO, and the samples’ absorbances were measured in a microplate reader (570 nm). The absorbance obtained from the control cells, which did not come into contact with the samples, was considered as 100% cell viability. Controls were made with 2% DMSO to prove that the cells remain viable using this percentage of solvent and with 50% DMSO as a control for cytotoxicity. The percentage of cell viability was expressed as the mean of the triplicates and its standard deviation using GraphPad Prism 8.0.1 software.

#### 2.5.4 Anti-Inflammatory Assay

The production of nitric oxide by RAW 264.7 macrophages was assessed indirectly through the quantification of nitrite in the cell culture medium using the Griess reaction.^40,41^ RAW 264.7 macrophages were distributed into 96-well plates in accordance with the cell viability assay protocol and were subsequently incubated at 37 °C with 5% CO_2_ in the course of a 24 h duration. After that, they were treated with the samples EEPV-F or EEPV-Q at noncytotoxic concentrations dissolved in DMSO at 2% and stimulated or not with lipopolysaccharide (LPS) (10 μg mL^–1^) and interferon γ (IFN-γ) (100 ng mL^–1^). Following a 24 h incubation period, 50 μL of the resulting fluid were subjected to a reaction with an equal volume of Griess reagents. Subsequent to a 15 min period of incubation, the samples were measured in a reader of microplates at 570 nm. The experiment was replicated thrice. The determination of nitrite concentrations was achieved through the use of a standard curve, which was constructed by using various concentrations of sodium nitrite. The results were expressed as nanomolar (nmol L^–1^).

### 2.6 Chemical Characterization of the Green Propolis Extract

The chemical profile of the propolis extracts (EEPV-F and EEPV-Q) was characterized through analyses of liquid chromatography coupled with mass spectrometry (LC- MS). In order to achieve this objective, solutions of the extracts in acetonitrile were prepared at a concentration of 200 ppm. These solutions were then filtered through syringe filters with a nylon membrane (0.45 μm). A Zorbax Eclipse C18 column (2.1 × 50 mm, 1.8 μm) maintained at 30 °C was used for chromatographic separations with a 3 μL injection volume. The elution process was conducted in gradient mode, with a flow rate of 0.350 mL min^–1^, utilizing a system comprising eluents A (H_2_O acidified with 0.1% formic acid) and B (acetonitrile acidified with 0.1% formic acid), in the following proportion: The initial dosage of B was between 5 and 100% of the total dosage, with this administered over a period of 22 min. This was followed by a second administration of B, ranging from 100 to 5% of the total dosage, which was administered over a period of 3 min. The MS1 and MS2 data were obtained using Agilent 6545 QTOF equipment, in both negative and positive mode, at three energy levels: low (capillary voltage: 2200 V, fragmentation voltage: 110 V, nozzle voltage: 300 V), medium (capillary voltage: 2500 V, fragmentation voltage: 120 V, nozzle voltage: 500 V) and high (capillary voltage: 3000 V, fragmentation voltage: 130 V, nozzle voltage: 600 V). The Auto MS/MS mode was executed by using a collision energy table with the following values: The molecular weight range is from 150 to 500 Da (20–25 electronvolts [eV]), from 500 to 1000 Da (25–50 eV), and from 1000 to 1500 Da (50–60 eV).^42^

The software MS-Dial 4.60 was used to perform the preprocessing of the data, which were subjected to analyses through the GNPS platform (Global Natural Products Network), the MS-Finder software, and the MSP library available for MS-Dial in order to perform the comparison of the MS2 spectra and subsequent annotation of the substances.

### 2.7 Prediction of Biological Activity

Aiming to predict the antiviral potential, the compounds identified through the application of liquid chromatography in conjunction with mass spectrometry were subsequently subjected to the prediction of biological activity analysis, utilizing the Prediction of Activity Spectra for Substances (PASS online) platform. A comparison was made between the structure of the substance and those of other active substances against the virus, as documented in the database. The decision was taken to focus on the action of the Rhinovirus and the Picornavirus because they both belong to the same group of coronaviruses (ssRNA—Group IV).^43^ The documented probabilities of each compound being active (Pa) and inactive (Pi) were then collated, and the compounds that showed Pa–Pi results ≥0.5 in at least one of the viruses were selected for subsequent analysis.^44^

### 2.8 Molecular Docking Analysis

In the preceding step, a selection of compounds was made that were then analyzed using molecular docking. This analysis was conducted with the AutoDock Vina tool and PyRx software, with the aim of understanding the interaction between the compounds and the target proteins in the fight against SARS-CoV-2.^45^ Crystal structures of the papain-like protease (PLpro) (Protomer PDB ID 7TZJ) of SARS-CoV-2 (2019-nCoV), spike glycoprotein (Protomer PDB ID 7QUS) and RNA-dependent RNA polymerase (RdRp) (PDB ID 8GY6) were obtained from the protein database (PDB). The selection of PDB files was predicated on considering SARS-CoV-2 as the source and the existence of a crystallographic ligand. Initially, the protein structure was prepared for docking by removing all water molecules and bound ligands from the protein using Biovia Discovery Studio software (San Diego, USA). The 3D structure of the selected compounds and controls (formoterol, arbidol, and remdesivir) was obtained from the PubChem database.^46–49^ The ligands were prepared by energy minimization and converted to AutoDock Ligand format (PDBQT) using the Open Babel module in the PyRx tool. A grid box of *X*: 22.0368, *Y*: 21.9009, *Z*: 23.9214 Å was at least defined to cover the active binding site: center *X*: 22.0368 *Y*: 21.9009 *Z*: 23.9214 for PLpro, *X*: 165.8895 *Y*: 163.9272 *Z*: 122.7481 for Spike and *X*: 62.0402, *Y*: 80.7382, *Z*: 91.6462 for RdRp. Then, an algorithm method was used to calculate the binding energies between the targets and the compounds by using the PyRx docking tool.

In addition, the objective was to validate the parameters of the docking approach. To this end, the crystallized ligands (S88 for PLpro, linoleic acid for Spike and gossypol for RdRp) were redocked into the binding pocket. The docked complex was superimposed onto the X-ray-resolved crystal bearing the cocrystallized ligand, with the aim of computing the root-mean-square deviation (RMSD) value. This was achieved using Biovia Discovery Studio software.

### 2.9 Statistical Analysis

The results were obtained from three measurements. All linear regressions in this work were analyzed by professional software Origin 6.0. The RAW 264.7 cell assays were examined through the utilization of a one-way analysis of variance (ANOVA), followed by a Dunnett’s post-test. The results were reported as the mean ± standard deviation, and GraphPad Prism 8 software was utilized. The test was also performed for the nitric oxide results, investigating the differences in the samples compared to the stimulated and untreated control groups. The analysis statistical for the hemolysis assay was analyzed through two-way ANOVA followed by Tukey’s post-test. The differences were considered significant when *p* ≤ 0.05.^50^

## 3 Results and Discussion

### 3.1 Yield of Extraction of the Dry Ethanolic Extract of Propolis (EEPV-F and EEPV- Q)

The influence of the extraction temperature was studied. The yields of extraction were 36.74% and 53.54% for extractions at room temperature and under heat, respectively (EEPV-F and EEPV-Q, respectively). The EEPV-Q sample showed the highest extraction efficiency, which can be attributed to the action of the temperature. Similar results were found by Kubiliene et al.^51^ and Oldoni et al.^52^ who demonstrated that the ideal temperature for a short extraction time and maximum yield of active constituents was 70 °C. Nonetheless, it is imperative to exercise caution when employing this method as prolonged exposure to elevated temperatures has the potential to compromise the integrity of thermolabile compounds within propolis and concomitantly augment the solubility of its cereal constituents.^53–55^ This justifies the chemical and cytotoxicity studies conducted in this work.

### 3.2 Evaluation of Antioxidant Activity and Determination of the Content of Phenolics and Total Flavonoids

The DPPH and ABTS assays are based on the same principle. In both cases, a synthetic colored radical or redox-active compound is generated. The ability of a biological sample to eliminate the radical or reduce the redox-active compound is then monitored by a spectrophotometer. As delineated in the specification, the ABTS assay is predicated on the generation of a blue/green ABTS^•+^ that can be reduced by antioxidants. Conversely, the DPPH assay is based on the reduction of purple DPPH^•^ to 1,1-diphenyl-2-picrylhydrazine. A higher DPPH^•^ radical scavenging activity and ABTS^•+^ are associated with a lower IC_50_, with such value being widely accepted as the concentration of the extract required to reduce the initial absorbance of the solution by 50%. It is evident that both assays are characterized by their ease of application, which is reflected in their popularity.

Table 1 shows the antioxidant activity (DPPH and ABTS methods) and total content of phenolics and flavonoids from the EEPV-F and EEPV-Q samples.

**Table 1:** Antioxidant Activity, Total Phenolic, and Flavonoid Contents of the Samples EEPV-F and EEPV-Q^a^

samples	DPPH	ABTS	total phenolics (mg GAE g^–1^)^b^	total flavonoids (mg QE g^–1^)^c^
	IC_50_ (μg mL^–1^)	IC_50_ (μg mL^–1^)		
EEPV-F	18.0 ± 0.3	16.6 ± 0.02	44 ± 2	10.6 ± 0.9
EEPV-Q	16.6 ± 0.5	15.3 ± 0.2	66 ± 3	6.6 ± 0.2
control	0.3 ± 0.01^d^	0.24 ± 0.02^d^		

a Results expressed as mean ± standard deviation.

b mg of galic acid equivalents (GAE) per g of sample/.

c mg of QE per g of sample/.

d Control quercetin.

The antioxidant activity (IC_50_) of the extracts measured by the DPPH and ABTS was similar, highlighting the potency of both methods to assess the antioxidant activity of the extract, and showed high antioxidant activity. These results indicate that the antioxidant activity and total flavonoid content were higher in the extraction performed at room temperature. The level of antioxidant capacity is directly correlated with the total content of phenolics and flavonoids. Despite the extant literature indicating that high temperatures can cause the breakdown of phenolics and flavonoids, the present study suggests that the phenolics were less affected. The thermal stability of phenolics and flavonoids is low, with this property primarily dependent on their structural characteristics, subclass, molecular weight, glycosylation, and esterification.^56^ Degradation is contingent on the structural solidity. Consequently, the dissociation of a double bond is accompanied by a greater requirement for energy. It has been demonstrated that alterations in the structural configuration of a given system invariably result in concomitant changes in the antioxidant activity. Moreover, it has been demonstrated that the process of degradation, initiated by elevated temperatures, can yield byproducts. The following observations were made: first, there was a decrease in antioxidant activity; second, the antioxidant activity of native flavonoids remained unchanged; and third, there was an increase in antioxidant activity. Our results were different from those obtained by Machado et al.^57^ Although the ethanolic extract of green propolis from different regions of Brazil had also been evaluated, the extraction process used was different from the one we employed. They found higher concentrations of phenolics (from 111.33 to 181.71 mg EAG g^–1^) and flavonoids (from 24.52 to 46.80 mg EQ g^–1^) but lower antioxidant activity (DPPH IC_50_ from 31.80 to 157.39). Cottica et al.^58^ also quantified phenolics, flavonoids, and antioxidant activity of compounds from the ethanolic extracts of Brazilian propolis from the state of Paraná. Despite the detection of comparable outcomes for phenolics (ranging from 48 to 87 mg EAG g^–1^ extract of propolis) and flavonoids (ranging from 10 to 26 mg EQ g^–1^ extract of propolis), the observed antioxidant activity values (IC_50_ ranging from 47 to 160) were found to be suboptimal in comparison to the previously reported values.

Although those authors reported results that differ from those presented in this study, other studies have shown similar findings. Thus, our results are consistent with previous studies reporting IC_50_ values for green propolis ranging from 15.0 to 20.0 μg mL^–1^ in DPPH assays^59^ and 14.5 to 18.0 μg mL^–1^ in ABTS assays, highlighting the potent antioxidant capacity of green propolis attributed to its rich phenolic and flavonoid content.^60^

Consequently, the outcomes may be subject to variation depending on the collection site and the species. The geographical provenance of the samples has been demonstrated to exert a significant influence on the diversity of the polyphenol content in propolis. This, in turn, has been shown to impact the biological activity and pharmacological effects observed among species.^61,62^

The significant antioxidant activity of green propolis observed in this study may represent a promising therapeutic strategy, particularly in the context of COVID-19. Oxidative stress is recognized as a key factor in the pathophysiology of COVID-19, contributing to excessive inflammation, immune dysregulation, and tissue injury. In this regard, previous studies have indicated that the antioxidant properties of propolis can help reduce the production of reactive oxygen species, mitigating inflammatory processes and supporting tissue repair mechanisms—all of which are essential for improving outcomes in viral infections such as COVID-19.^63^

### 3.3 Evaluation of the Cytotoxicity and Anti-Inflammatory Activity of the Extracts

Before conducting the assay to evaluate the anti-inflammatory activity, we assessed the percentage of viability of RAW 264.7 macrophages following a 24 h exposure period to the extracts (Figure 1). The samples were considered noncytotoxic only when the viability of the macrophages was above 70%.^64^ The results indicate that only the highest concentration (500 μg mL^–1^) of EEPV-F demonstrated significant toxicity, suggesting a reduced safety profile against these cells in comparison to lower concentrations of EEPV-F and EEPV-Q at all concentrations evaluated. Many studies have already shown that different extracts of green propolis are biocompatible and noncytotoxic. As an example, a green propolis extract obtained commercially (Pharma Nectar, Brazil) has been reported as nontoxic to the cells BMMSC (mesenchymal stromal cells) and induced their proliferation.^65^ The same study suggested that green propolis extract may enhance tissue regeneration. Another study demonstrated that Brazilian green propolis rescued the viability losses of HUVEC cells (human umbilical vein endothelial cells) and that still was able to protect them against oxidative damage.^66^ The findings of our study corroborate what is found in the literature, showing that propolis green derivatives can often be considered safe for various purposes, including the investigation of anti-inflammatory activity.

**Figure 1:**
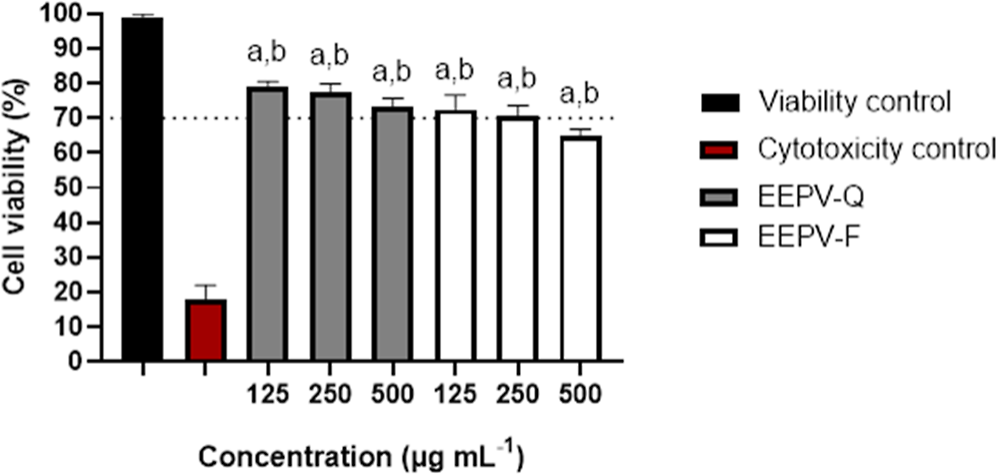
Evaluation of the cytotoxicity of EEPV-F and EEPV-Q samples. Statistical treatment: (a) statistical difference compared to the viability control; (b) statistical difference compared to the cytotoxicity control. The differences were considered significant when *p* < 0.05.

After assessing cell viability, nitric oxide (NO) production was induced in RAW 264.7 macrophages as NO has pro-inflammatory effects that can lead to tissue destruction and even worsen the course of COVID-19 and lead to death.^67^ Figure 2 shows the nitrite concentration after treatment with EEPV-Q and EEPV-F. A reduction in nitrite levels is observed at all concentrations of EEPV-Q, especially at the highest. EEPV-F on the other hand showed a reduction in nitrite levels only at 250 μg mL^–1^.

**Figure 2:**
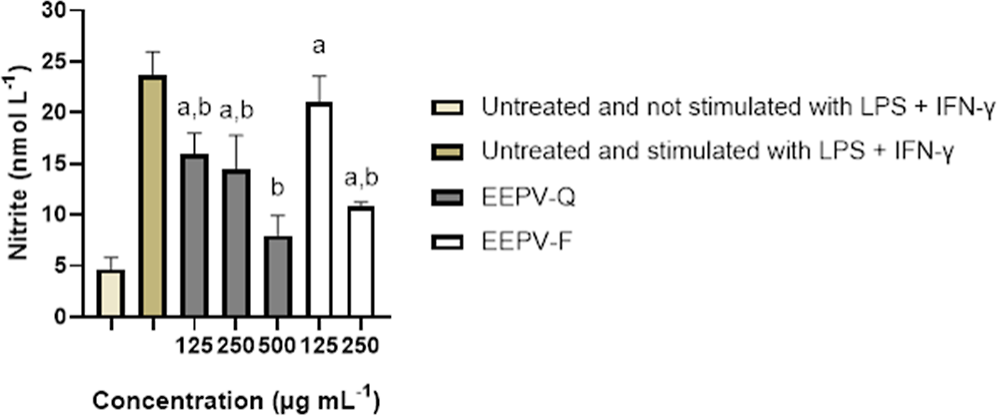
Levels of nitrite in macrophages in the positive control group (untreated and not stimulated with LPS + IFN-γ), negative control (untreated macrophages stimulated with LPS + IFN-γ), and after treatment with EEPV-F and EEPV-Q. The results represent the mean ± the standard deviation of the triplicates of the experiments. (a) Statistical difference in relation to the untreated and unstimulated LPS + IFN-γ control; (b) statistical analysis revealed a discrepancy in relation to the untreated and stimulated LPS + IFN-γ control.

The present study corroborates the findings of another study, which demonstrated a reduction in NO levels caused by the hydroalcoholic extract of Brazilian green propolis.^68^ Furthermore, it has already been demonstrated that the addition of propolis to the treatment of hospitalized patients with COVD-19 resulted in clinical benefits, especially with regard to reducing the length of hospitalization.^69^ Our results, in addition to those found in the literature, suggest that the reducing effect of NO caused by propolis may be associated with clinical improvement in patients with COVID-19.

### 3.4 Assessment of Hemolytic Activity

Cytotoxicity studies are of paramount importance during the characterization of novel compounds or materials that are intended for use within human biological systems *in vivo*. In this study, the hemocompatibility of different concentrations of samples EEPV-F or EEPV-Q was evaluated through a hemolysis test to determine their hemolytic potential. The term hemolysis refers to the release of hemoglobin into the surrounding fluid (blood plasma or normal saline) due to the disruption (lysis) of the erythrocyte membrane *in vivo* (general blood circulation) or *in vitro* (deionized water). It is understood that this was the inaugural instance in which the hemolysis test was conducted using a propolis extract.

To evaluate the integrity of the erythrocyte membrane under exposure to increasing doses of EEPV-F and EEPV-Q, hemolytic activity assessments were performed *in vitro* in two stages using defibrinated sheep blood. Figure 3 presents the percentage of hemolysis following treatment with EEPV-F and EEPV-Q at different concentrations and incubation times. The hemolytic potential of both extracts was determined by incubating erythrocytes isolated from sheep.

**Figure 3:**
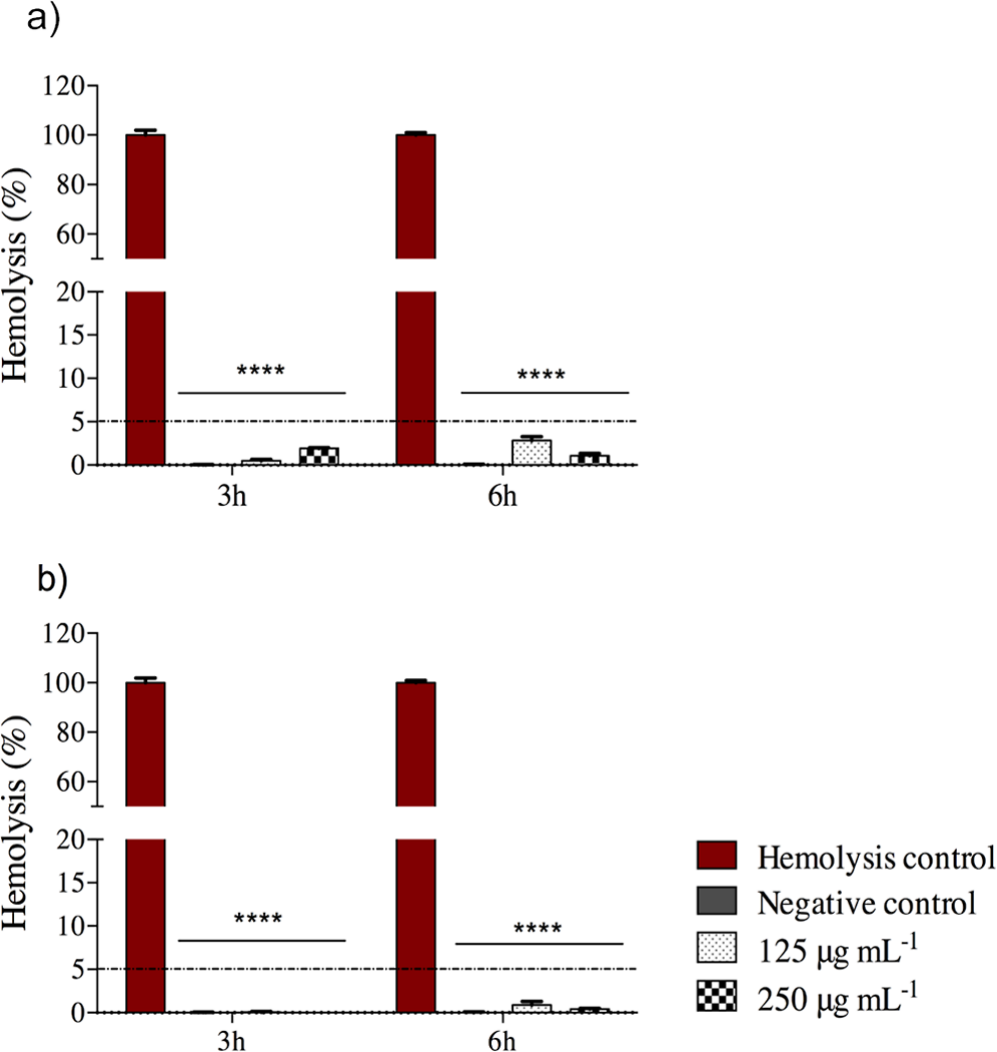
Hemolytic percentage of samples exposed to EEPV-Q (a) and EEPV-F (b), respectively. The assays were conducted using bovine erythrocytes at two different concentrations (125 and 250 μg mL^–1^) and at two incubation times (3 and 6 h). 1x PBS and Milli-Q water were used as negative control and hemolysis control, respectively. Statistical significance (****p < 0.0001 vs hemolysis control) was determined by two-way ANOVA followed by Tukey’s post-test.

Notably, all concentrations (125 and 250 μg mL^–1^) of EEPV-F and EEPV-Q exhibited hemolytic activity below 5% compared to the positive control (deionized water, hypotonic solution) after 3 and 6 h of incubation. The percentages obtained from our analysis are consistent with the recommended and established thresholds outlined in ASTM F756–17, the standard practice for evaluating the *in vitro* hemolytic properties of materials intended for use in contact with blood.^70^ Supporting our findings, Wozniak et al.^71^ demonstrated that propolis extract can protect human red blood cells against free radicals, exhibiting cytoprotective activity and low activity hemolytic. Considering that hemocompatibility is a critical parameter not only for systemic safety but also in contexts such as reproductive health, where recent studies have explored potential associations between viral infections and early pregnancy outcomes, our findings reinforce the importance of evaluating natural compounds with potential biomedical applications.^72^

### 3.5 Chemical Characterization of the Green Propolis Extract

In the context of tropical countries, Brazil has been identified as a region of particular significance due to its chemical diversity, especially regarding the types of propolis present. Furthermore, Brazilian green propolis is the most widely produced and consumed both internally and externally. The substance under scrutiny is characterized by its abundance of phenylpropanoids, prenylated phenylpropanoids (a class that includes artepillin C), and sesqui- and diterpenoids. The material under investigation is obtained from the tips of *Baccharis dracunculifolia* and/or green propolis (Asteraceae).^73^ Thus, Salatino et al.^74^ detected phenolic compounds, many of which were prenylated. It has been posited that such substances are constituents of the *B. dracunculifolia* and/or Brazilian green propolis: 1, 3, 5, 9, 10, 14, 24, 25, 28, and 35, thus designated given the 52 compounds identified for such species. Moura et al.^75^ identified several compounds, which are acids caffeoylquinic, comprising 1–3 residues of caffeic acid (caffeoylquinic acid, tricaffeoylquinic acid, dicaffeoylquinic acid, caffeoylferuloylquinic acid, and tricaffeoylquinic acid). It is evident that such compounds are characteristic and abundant constituents of green propolis, which is ideally obtained from an aqueous extract. Likewise, in this study, compounds such as caffeic acid were also identified (Table 2), as well as some of its isomers being identified. Luteolin –O -glucuronide—has also been identified by Ferreres et al.,^76^ but glycosylated as well as dimethoxy- naringenin-diglycoside were identified in the genotype of citrus. In addition, Refaat et al.^77^ highlighted compounds from propolis, especially quercetin and naringin which were also identified in this research. Finally, compounds such as 4-hydroxybenzoic acid, *p*-coumaric acid, ferulic acid, pinocembrin, luteolin, kaempferol, and kaempferide were identified by Peter et al.^78^ Consequently, the present study has identified novel compounds that have not yet been documented within the existing body of literature. These compounds may offer significant potential in the fight against anti-Sars-Cov-2. Thus, it is possible to note the studied compounds and their relevance to this study in Table 2.

**Table 2:**
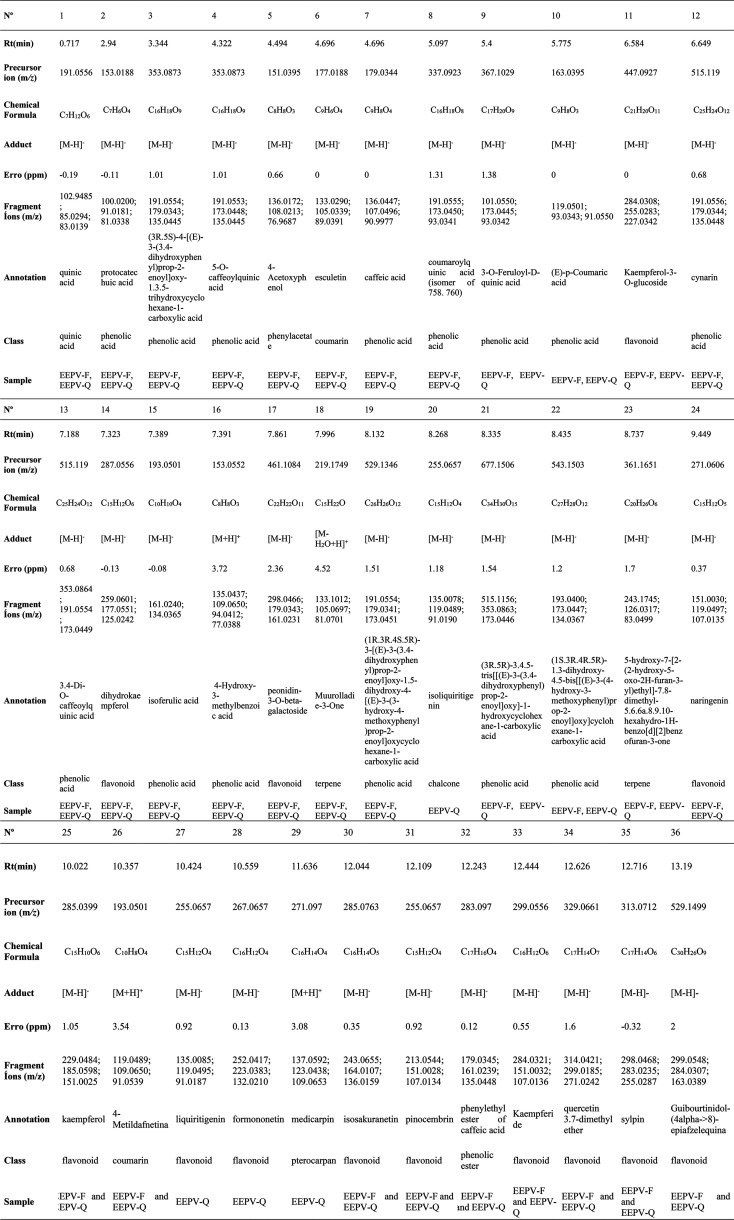

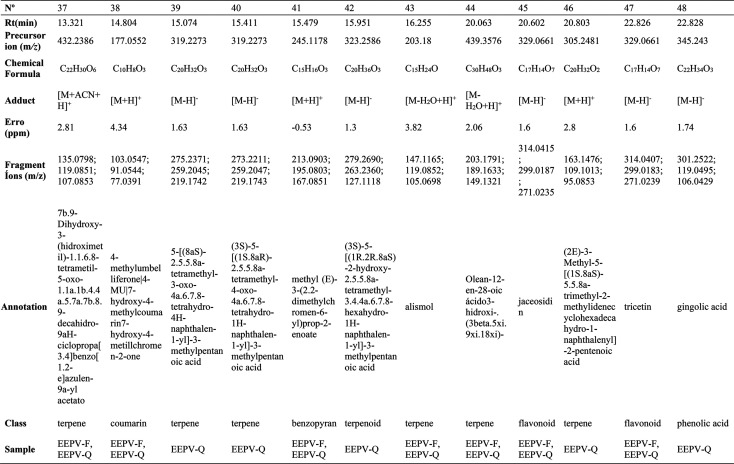
Compounds from the Ethanolic Extract of Green Propolis

### 3.6 Prediction of Biological Activity

Antiviral data for SARS-CoV-2 are unavailable on the platform used (PASSonline) since COVID-19 is a relatively recent viral disease. Therefore, in this study in silico, a screening was performed with the identified compounds in EEPV-F and EEPV-Q, for antiviral activity against rhinovirus and picornavirus, which belong to the same group as the coronavirus (Sars-Cov-2) since coronavirus is not yet included in the database. It included analysis of the potential as inhibitors of the 3CL-protease enzyme as it is involved in the infection cycle of Sars-Cov-2. This enzyme processes the polyproteins that entered the cells, in order to lead them to proteins nonstructural, which mediate the process of genome replication.^43^

The results obtained in the PASSonline are presented in Table 3. The analysis revealed that none of the compounds under investigation exhibited any potential for the 3CL-protease enzyme. Conversely, 18 compounds demonstrated notable antiviral activity against rhinovirus and picornavirus. The selection of compounds for analysis by molecular docking was based on their higher likelihood of being active, as indicated by a Pa-Pi ≥ 0.5 for at least one of the two analyzed viruses.

**Table 3:** Prediction of Biological Activity of the Compounds from the Ethanolic Extract of Green Propolis

compound	antiviral (picornavirus)			antiviral (rhinovirus)			3C-like protease		
	Pa	Pi	Pa-Pi	Pa	Pi	Pa-Pi	Pa	Pi	Pa-Pi
quinic acid	0.615	0.016	0.6	0.557	0.011	0.5	0.257	0.066	0.191
protocatechuic acid	0.588	0.022	0.6	0.489	0.029	0.5	0.37	0.005	0.365
(3R,5S)-4-[(E)-3-(3,4-dihydroxyphenyl)prop-2-enoyl]oxy-1,3,5-trihydroxycyclohexane-1-carboxylic acid	0.4	0.11	0.3	0.483	0.031	0.5			
5-O-caffeoylquinic acid	0.433	0.085	0.3	0.472	0.036	0.4			
4-acetoxiphenol	0.628	0.014	0.6	0.519	0.019	0.5	0.283	0.037	0.246
Esculetin	0.378	0.129	0.2				0.22	0.13	0.09
caffeic acid	0.508	0.047	0.5	0.389	0.103	0.3	0.218	0.134	0.084
coumaroyl quinic acid (isomer of 758, 760)	0.437	0.083	0.4	0.483	0.031	0.5			
3-*O*-Feruloyl-D-quinic acid	0.348	0.161	0.2	0.455	0.046	0.4			
(E)-*p*-coumaric acid	0.545	0.034	0.5	0.384	0.109	0.3	0.235	0.101	0.134
kaempferol-3-*O*-glucoside									
cinarin	0.377	0.13	0.2	0.472	0.036	0.4			
3,4-di-O-caffeoylquinic acid	0.433	0.085	0.3						
dihydrokaempferol	0.322	0.194	0.1	0.528	0.016	0.5	0.198	0.177	0.021
isoferulic acid	0.412	0.1	0.3				0.2	0.173	0.027
4-hydroxy-3-methylbenzoic acid	0.519	0.043	0.5	0.451	0.048	0.4	0.359	0.006	0.353
peonidin-3-O-beta-galactoside									
muurolladie-3-one				0.46	0.042	0.4			
(1R,3R,4S,5R)-3-[(E)-3-(3,4-dihydroxyphenyl)prop-2-enoyl]oxy-1,5-dihydroxy-4-[(E)-3-(3-hydroxy-4-methoxyphenyl)prop-2-enoyl]oxycyclohexzane-1-carboxylic acid	0.3	0.227	0.1	0.462	0.042	0.4			
isoliquiritigenin	0.302	0.224	0.1				0.222	0.124	0.098
(3R,5R)-3,4,5-tris[[(E)-3-(3,4-dihydroxyphenyl)prop-2-enoyl]oxy]-1-hydroxycyclohexane-1-carboxylic acid	0.452	0.074	0.4	0.355	0.022	0.3			
(1S,3R,4R,5R)-1,3-dihydroxy-4,5-bis[[(E)-3-(4-hydroxy-3-methoxyphenyl)prop-2-enoyl]oxy]cyclohexane-1-carboxylic acid	0.348	0.161	0.2	0.455	0.046	0.4			
5-hydroxy-7-[2-(2-hydroxy-5-oxo-2H-furan-3-yl)ethyl]-7,8-dimethyl-5,6,6a,8,9,10-hexahydro-1H-benzo[d][2]benzofuran-3-one									
naringenin	0.3	0.227	0.1	0.611	0.005	0.6			
kaempferol	0.322	0.194	0.1	0.528	0.016	0.5	0.198	0.177	0.021
4-methyldaphnetin	0.368	0.139	0.2	0.388	0.104	0.3	0.218	0.133	0.085
liquiritigenin	0.363	0.143	0.2	0.632	0.004	0.6	0.222	0.126	0.096
formononetin	0.296	0.235	0.1	0.35	0.159	0.2	0.227	0.116	0.111
medicarpin	0.307	0.216	0.1	0.477	0.034	0.4	0.236	0.1	0.136
isosakuranetin				0.593	0.007	0.6			
pinocembrin	0.314	0.205	0.1	0.608	0.005	0.6	0.196	0.183	0.013
caffeic acid phenethyl ester	0.44	0.081	0.4	0.415	0.075	0.3	0.057	0.008	0.049
kaempferide				0.516	0.02	0.5			
quercetin 3,7-dimethyl éter				0.296	0.261	0.0	0.192	0.19	0.002
sylpin							0.219	0.131	0.088
guibourtinidol-(4alpha→8)-epicatechin				0.482	0.032	0.5			
7b,9-dihydroxy-3-(hidroximetil)-1,1,6,8-tetrametil-5-oxo-1,1a,1b,4,4a,5,7a,7b,8,9-decahidro-9aH-ciclopropa[3,4]benzo[1,2-e]azulen-9a-yl acetato				0.384	0.11	0.3			
4-metilumbelliferone|4-MU|7-Hidroxi-4-metilcoumarina7-hidroxi-4-metillchromen-2-one									
5-[(8aS)-2,5,5,8a-tetramethyl-3-oxo-4a,6,7,8-tetrahydro-4H-naphthalen-1-yl]-3-methylpentanoic acid	0.292	0.241	0.1	0.424	0.068	0.4			
(3S)-5-[(1S,8aR)-2,5,5,8a-tetramethyl-4-oxo-4a,6,7,8-tetrahiro-1H-naphthalen-1-yl]-3-methylpentanoic acid				0.377	0.119	0.3			
metil (E)-3-(2,2-dimetillchromen-6-il)prop-2-enoato				0.336	0.183	0.2			
(3S)-5-[(1R,2R,8aS)-2-hydroxy-2,5,5,8a-tetramethyl-3,4,4a,6,7,8-hexahydro-1H-naphthalen-1-yl]-3-methylpentanoic acid	0.315	0.204	0.1	0.426	0.066	0.4			
alismol				0.347	0.164	0.2			
olean-12-en-28-oic ácido3-hidroxi-, (3beta,5xi,9xi,18xi)-				0.481	0.032	0.4	0.35	0.007	0.343
jaceosidin				0.296	0.26	0.0	0.218	0.133	0.085
(2E)-3-methyl-5-[(1S,8aS)-5,5,8a-trimethyl-2-methylenedecahydro-1-naphthalenyl]-2-pentenoic acid									
tricin				0.571	0.009	0.6			
glycolic acid	0.458	0.071	0.4				0.199	0.176	0.023

### 3.7 Molecular Docking Analysis

The results of redocking and superimposition indicated that the docking protocol used can be considered as efficient and valid due to low RMSD values and similar interactions compared to the native cocrystallized complexes (Figure 4). The results of the green propolis compounds for the enzymes PLpro (7TZJ), RdRp (8GY6), and spike (7QUS) are presented in Table 4.

**Figure 4:**
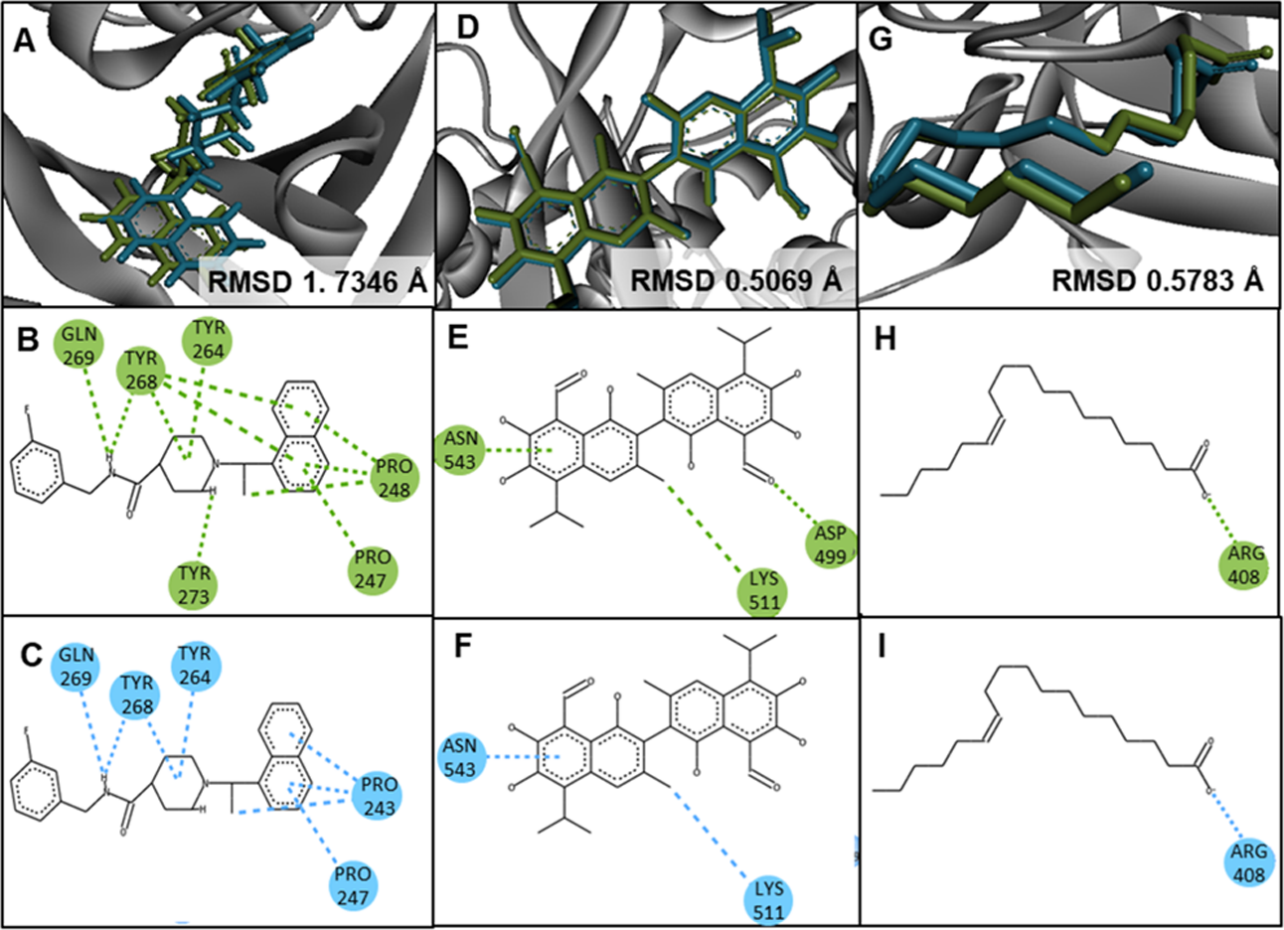
Following methodology was employed: first, redocked structures were superimposed, and second, a two-dimensional molecular interaction analysis was performed for PLpro (A–C), RdRp (D–F), and spike (G–I). As illustrated, the cocrystallized ligand is represented by the color green, while the redocked ligand is indicated by the color blue.

**Table 4:** Molecular Docking Analysis Performed for the Enzymes PLpro, RdRp, and Spike

compound name	binding affinity (kcal mol^–1^)		
	PLpro	RdRp	Spike
quinic acid	–4.0	–6.6	–4.4
protocatechuic acid	–4.2	–6.9	–4.2
acid (1r,3R,4s,5S)-4-{[(2E)-3-(3,4-Dihydroxyphenyl)-2-propenoyl]oxi}-1,3,5- trihydroxycyclohexanecarboxylic	–5.9	–6.1	–5.9
4-acetoxyphenol	–4.2	–5.9	–4.0
caffeic acid	–4.9	–6.8	–5.0
coumaroyl quinic acid (isomer of 758, 760)	–5.7	–6.8	–5.8
(E)-p-coumaric acid	–4.7	–6.5	–4.8
dihydrokaempferol	–5.3	–6.8	–5.8
4-hydroxy-3-methylbenzoic acid	–4.3	–6.2	–4.2
naringenin	–6.0	–7.0	–6.1
kaempferol	–5.4	–7.0	–5.8
liquiritigenin	–5.9	–7.2	–5.9
isosakuranetin	–6.2	–7.1	–6.0
pinocembrin	–6.0	–6.9	–6.1
kaempferide	–5.4	–7.0	–5.6
guibourtinidol-(4alpha→8)-epicatechin	–6.0	–8.5	–6.9
tricin	–5.8	–7.0	–6.2
positive control	–6.0	–7.0	–5.2
	formoterol	remdesivir	arbidol

The PLpro enzyme is responsible for playing a vital role in transcription/replication in the coronavirus cycle. The enzyme in question demonstrated a higher degree of binding affinity (lower binding energy) for compounds including naringenin, isosakuranetin, pinocembrin, and guibourtinidol (4alpha→8)-epicatechin than that for the positive control, formoterol. Formoterol is a drug used as a bronchodilator β adrenergic 2-agonists as one of the main medications for asthma and has already been tested for SARS-CoV-2, in order to verify its antiviral action.^79^

In addition to the initial enzyme, the compounds guibourtinidol-(4alpha→8)-epicatechin, naringenin, kaempferol, liquiritigenin, isosakuranetin, kaempferide, and tricin exhibited a comparable or greater binding affinity in comparison to the positive control remdesivir, as determined by the RNA-dependent RNA polymerase (RdRp) assay. This drug is used for the treatment of viruses as an RdRp inhibitor. Thus, it can inhibit the virus by inhibiting the synthesis of viral nucleic acid since remdesivir can bind to the RNA-binding channel of SARS-CoV-2 RdRp.^80^

Finally, the spike protein must be considered. This is mediated by proteases such as TMPRSS2, which are enzymes that break down protein amino acids. They thus play an important role in viral infection. The compounds that showed the best results were acid (1r,3R,4s,5S)-4-{[(2E)-3-(3,4- dihydroxyphenyl)- 2-propenoic]­oxy} −1,3,5 trihydroxycyclohexanecarboxylic, coumaroyl quinic acid, dihydrokaempferol, naringenin, kaempferol, dihydrokaempferol, liquiritigenin, isosakuranetin, pinocembrin, kaempferide, guibourtinidol-(4alpha→8)-epicatechin, and tricin. All have shown higher binding affinity than the positive control Arbidol. This is an antiviral drug which can inhibit the virus, in order to prevent the contact and penetration of the virus into host cells, avoiding the fusion of lipid envelope of the virus with the cell membrane. Zheng et al.^81^ showed to inhibit the process of infection by the virus responsible for the onset of the disease, by interfering with the release of the virus from intracellular vesicles.

Comparing all the results obtained by molecular docking, guibourtinidol-(4alpha→8)-epicatechin stands out for having the strongest affinity (lowest energy of binding) (Table 4). The interactions of this compound are mainly of the hydrogen bond and hydrophobic types (Figure 5). It has been suggested that the interaction of this compound with the enzymes may result in the prevention of these functions, which, in turn, may prevent the replication of SARS-CoV-2.^82–84^

**Figure 5:**
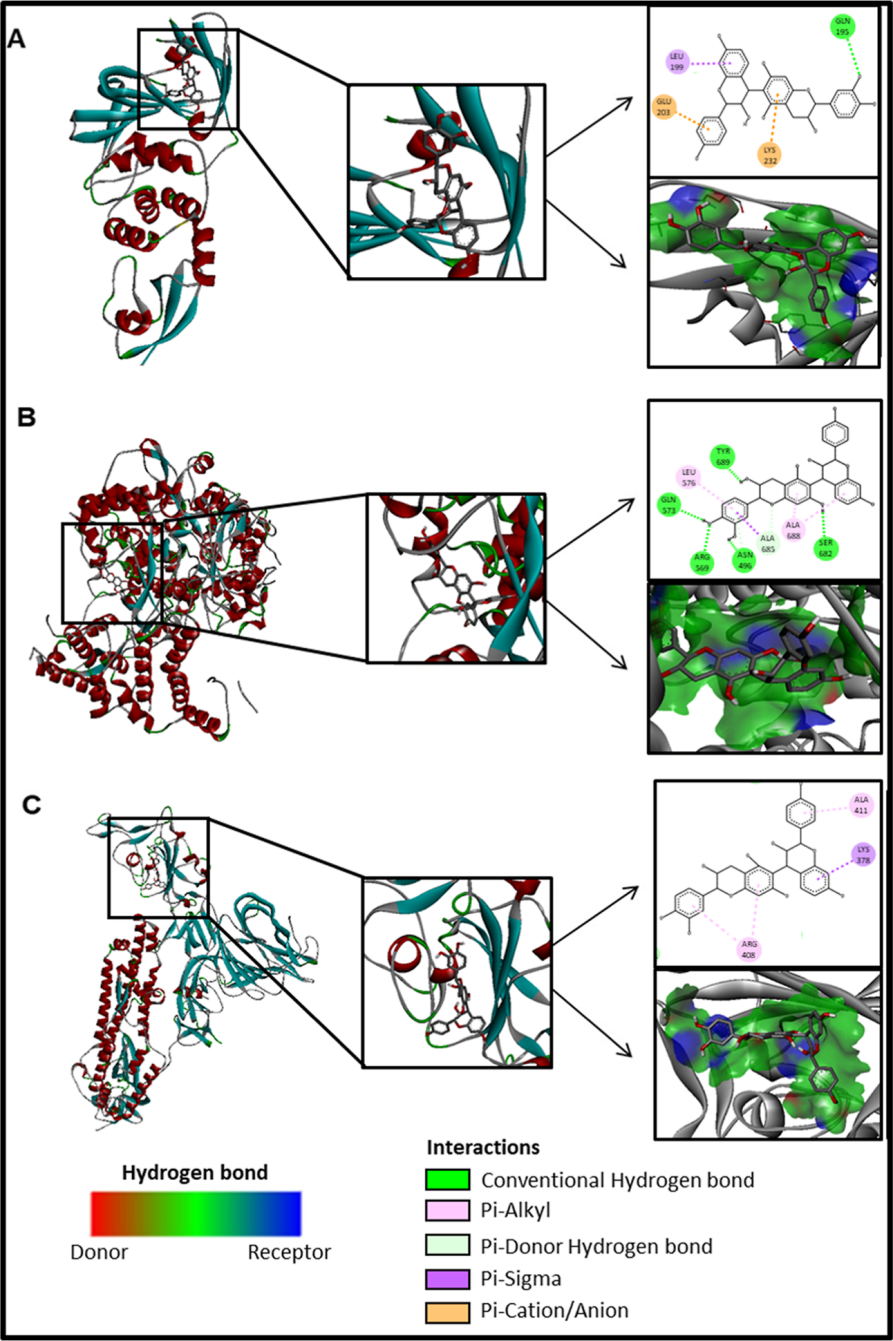
3D diagram and interaction diagrams of the compound guibourtinidol-(4alpha→8)-epicatechin with PLpro (A), RdRp (B), and protein S (C).

## 4 Final Considerations

The results obtained from the tests with ethanolic extract of green propolis, obtained at two different temperatures, demonstrated anti-inflammatory potential and low toxicity, which allows for its study with a view to the application for anti-Sars-Cov-2 treatment. With regard to the in silico evaluations, the aforementioned compounds exhibited potential for inhibition of action targets against the virus. Consequently, the present research has demonstrated the potential of the studied green propolis extracts in the treatment of the symptoms of the COVID-19 disease and has laid the foundation for future studies to explore new applications.
